# Parotitis Induced by Botulinum‐Toxin Injections to the Masseter Muscle

**DOI:** 10.1111/jocd.70052

**Published:** 2025-02-12

**Authors:** Nadav Grinberg, Oren Peleg

**Affiliations:** ^1^ Department of Otolaryngology, Head and Neck Surgery and Maxillofacial Surgery Tel‐Aviv Sourasky Medical Center Tel Aviv Israel

We would like to thank Nishikawa et al. for their comprehensive review of adverse events following botulinum toxin (BTX) injections for treating masseter muscle hypertrophy [1]. While Nishikawa et al. [2] focused on adverse events such as paradoxical bulging, sunken cheeks, and facial asymmetry, we would like to propose parotitis as an additional adverse effect based on recent literature.

Research on the impact of BTX injections into masticatory muscles in the parotid gland is limited. While some reports observed no severe adverse effects or mild xerostomia accompanied by normal salivary flow rates [3], other studies report patients having reduced salivary volume and thicker saliva [4].

The proximity of the masseter muscle to the parotid gland suggests that any neuromodulator injection to this area could inadvertently affect the parotid gland. Several mechanisms have been proposed for this effect. Anatomically, the proximity of the injection site to critical structures could lead to inadvertent influence on salivary output [4]. Additionally, the complex and variable branching of the facial nerve, including the potential secretomotor branch, could be affected and thereby altering salvation [5]. Physical factors such as gravitational flow, injection pressure, or diffusion of the neuromodulator across fascial planes could further promote unintended BTX spread [5, 6, 7]. Reduced muscular support around the gland may also alter intraglandular pressure and decrease salivary flow [7]. Physiological factors, for instance, efficient local uptake or systemic neurotoxin spread may impair salivary production as well [8]. All of which may lead to hyposalivation, causing stasis in the gland and potential retrograde infection [9].

Parotitis induced by BTX injection, although rare, should be carefully considered by clinicians performing these procedures. Given the increasing use of BTX in both therapeutic and aesthetic practices, even rare complications may occur. When administering BTX, it is vital to pay attention to the accuracy of the injection site. Palpation should be used to delineate the bulk of the masseter muscle, targeting its hypertrophic region while avoiding the posterior border of the muscle [4]. The needle should be inserted perpendicularly. The appropriate injection depth has a crucial role as well. The needle should be inserted deep and encounter the ramus, minimizing the risk of invading the parotid gland [10]. In order to avoid unwanted results, two fundamental pillars need to be preserved. First, proper case selection is mandatory. Thorough anamnesis and medical history should be taken before considering any treatment plan. Second, adequate and continuous clinical training will improve the clinician's ability to perform the procedure. Additionally, when addressing highly anatomically complex areas, the use of monitoring tools such as ultrasound guidance or electromyographic monitoring is recommended [6, 11].

Future clinical research is needed to identify factors contributing to complications from BTX injections in facial procedures. By focusing on manageable factors, we can help reduce risks for our patients before, during, and after surgery.

## Conflicts of Interest

The authors declare no conflicts of interest.

## Data Availability

The data that support the findings of this study are available from the corresponding author upon reasonable request.
